# Health Professionals' Knowledge, Attitudes and Practices about Pharmacovigilance in India: A Systematic Review and Meta-Analysis

**DOI:** 10.1371/journal.pone.0152221

**Published:** 2016-03-24

**Authors:** Akshaya Srikanth Bhagavathula, Asim Ahmed Elnour, Shazia Qasim Jamshed, Abdulla Shehab

**Affiliations:** 1 Department of Clinical Pharmacy, University of Gondar-College of Medicine and Health Sciences, School of Pharmacy, Gondar, Ethiopia; 2 Department of Clinical Pharmacy, Faculty of Pharmacy, Fatima College of Heath Sciences, FCHS-Al Ain Campus, Al Ain, UAE; 3 Department of Pharmacy Practice, Kulliyyah of Pharmacy, International Islamic University Malaysia, Kuantan, Pahang, Malaysia; 4 Internal Medicine Department, College of Medicine and Health Sciences, UAE University, Al Ain, UAE; The Foundation for Medical Research, INDIA

## Abstract

**Background:**

Spontaneous or voluntary reporting of suspected adverse drug reactions (ADRs) is one of the vital roles of all health professionals. In India, under-reporting of ADRs by health professionals is recognized as one of the leading causes of poor ADR signal detection. Therefore, reviewing the literature can provide a better understanding of the status of knowledge, attitude and practice (KAP) of Pharmacovigilance (PV) activities by health professionals.

**Methods:**

A systematic review was performed through Pubmed, Scopus, Embase and Google Scholar scientific databases. Studies pertaining to KAP of PV and ADR reporting by Indian health professionals between January 2011 and July 2015 were included in a meta-analysis.

**Results:**

A total of 28 studies were included in the systematic review and 18 of them were selected for meta-analysis. Overall, 55.6% (95% CI 44.4–66.9; *p*<0.001) of the population studied were not aware of the existence of the Pharmacovigilance Programme in India (PvPI), and 31.9% (95% CI 16.3–47.4; *p*<0.001) thought that "all drugs available in the market are safe". Furthermore, 28.7% (95% CI 16.4–40.9; *p*<0.001) of them were not interested in reporting ADRs and 74.5%, (95% CI 67.9–81.9; *p*<0.001) never reported any ADR to PV centers.

**Conclusion:**

There was an enormous gap of KAP towards PV and ADR reporting, particularly PV practice in India. There is therefore an urgent need for educational awareness, simplification of the ADR reporting process, and implementation of imperative measures to practice PV among healthcare professionals. In order to understand the PV status, PvPI should procedurally assess the KAP of health professionals PV activities in India.

## Introduction

India is home to one of the largest drug consuming populations in the world. There are between 60,000–80,000 brands of drugs available in the Indian market that are irrationally prescribed and misused [1]. This may be due to lack of medication safety practices, and failures in the regulatory environment. The misuse and faulty prescribing account for considerable development of adverse drug reactions (ADRs) that are one of the major causes of mortality and morbidity, unplanned hospitalization, and increased healthcare cost, worldwide [2–5]. Thus, early identification of ADRs is extremely important for both government and non-government health care organizations.

The World Health Organization (WHO) defines ADRs as any noxious, unintended, and undesired effect of a drug, which occurs at doses used in humans for prophylaxis, diagnosis, or treatment of the disease [6]. The worldwide incidence of ADR occurrence leading to emergency hospitalization ranges from 0.2 to 41.3%, while 28.9% of these ADRs are preventable [7]. In 2012, a meta-analysis showed that 52% of ADR-related emergency hospitalizations and 45% of ADRs in inpatients were preventable [8]. Moreover, post-marketing safety studies have been shown to be very important in identifying possible risk factors associated with the use of new drugs in the general population and the contribution of health professionals is significant in reporting suspected ADRs to strengthen signal detection.

Pharmacovigilance (PV) is and the sum of activities related to the detection, assessment, understanding, and prevention of ADRs caused by drugs [9]. Spontaneous reporting of suspected ADRs to PV centers is of utmost importance to generate the safety data of marketed drugs. Indeed, understanding the importance of reporting ADRs, national and international organizations urged health professionals to prioritize ADR reporting in order to curtail ADR- related problems. In India, the national Pharmacovigilance Programme of India (PvPI) was established by the Central Drugs Standard Control Organization (CDSCO) in 2004 to monitor ADRs and to provide drug safety reports to the WHO-ADR monitoring center in Uppsala, Sweden [10]. To coordinate ADR monitoring throughout India, the Drug Controller General of India (DCGI) and Indian Council of Medical Research (ICMR) have established many peripheral PV centers in various hospitals located in major Indian cities [11].

Furthermore, it is evident that under-reporting of suspected ADRs by health professionals is a widespread problem in India [12]. For instance, the contribution of ADR reporting from India was below 1%, which highlights the existing gaps in success of the PV programme [13]. There are several local and national projects that are aimed at improving and promoting PV activities in India [14–18]. These initiatives seek to increase awareness of the PV programme among health professionals and to improve ADR reporting. In particular, a better understanding of these issues could help national organizations in developing strategies for improvement of PV activities. Hitherto, no systematic review on this topic was identified from India. In order to gather data from the existing evidence pertaining to knowledge, attitude and practice (KAP) of ADR reporting and PV in the health professionals, a systematic review of current literature and a meta-analysis were performed.

## Materials and Methods

To summarize the existing evidence related to PV activities in India, a systematic review using Preferred Reporting Items for Systematic Reviews and Meta-analysis (PRISMA) statements was conducted (S1 Appendix). Cross-sectional observational studies investigating the KAP of PV activities in India were considered. Papers that are original peer-reviewed research articles published in English from January 2011- July 2015 were retrieved from four databases: Pubmed, Scopus, Embase and Google Scholar. We limited our review to studies that used a structured questionnaire administered to Indian health professionals (doctors, nurses, dentists, pharmacists, medical students, postgraduate residents) assessing the following:

- Awareness of PvPI
- Knowledge about the safety of drugs
- Attitude towards reporting ADRs
- Knowledge about obtaining ADR forms
- Practice of ADR reporting
- Availability of ADR forms
- Surveys using open answers focused on health professionals

### Data collection

Titles and abstracts were screened by one author (ASB). Studies were selected for inclusion from full-text articles by two researchers (ASB, AAE). The database search fields for titles, abstracts and index terms were searched using the following research string: pharmacovigilance* AND adverse drug reactions reporting* AND health professionals* AND survey* AND India* AND (knowledge OR attitude OR practice).

### Selection of studies

**Fig 1:**
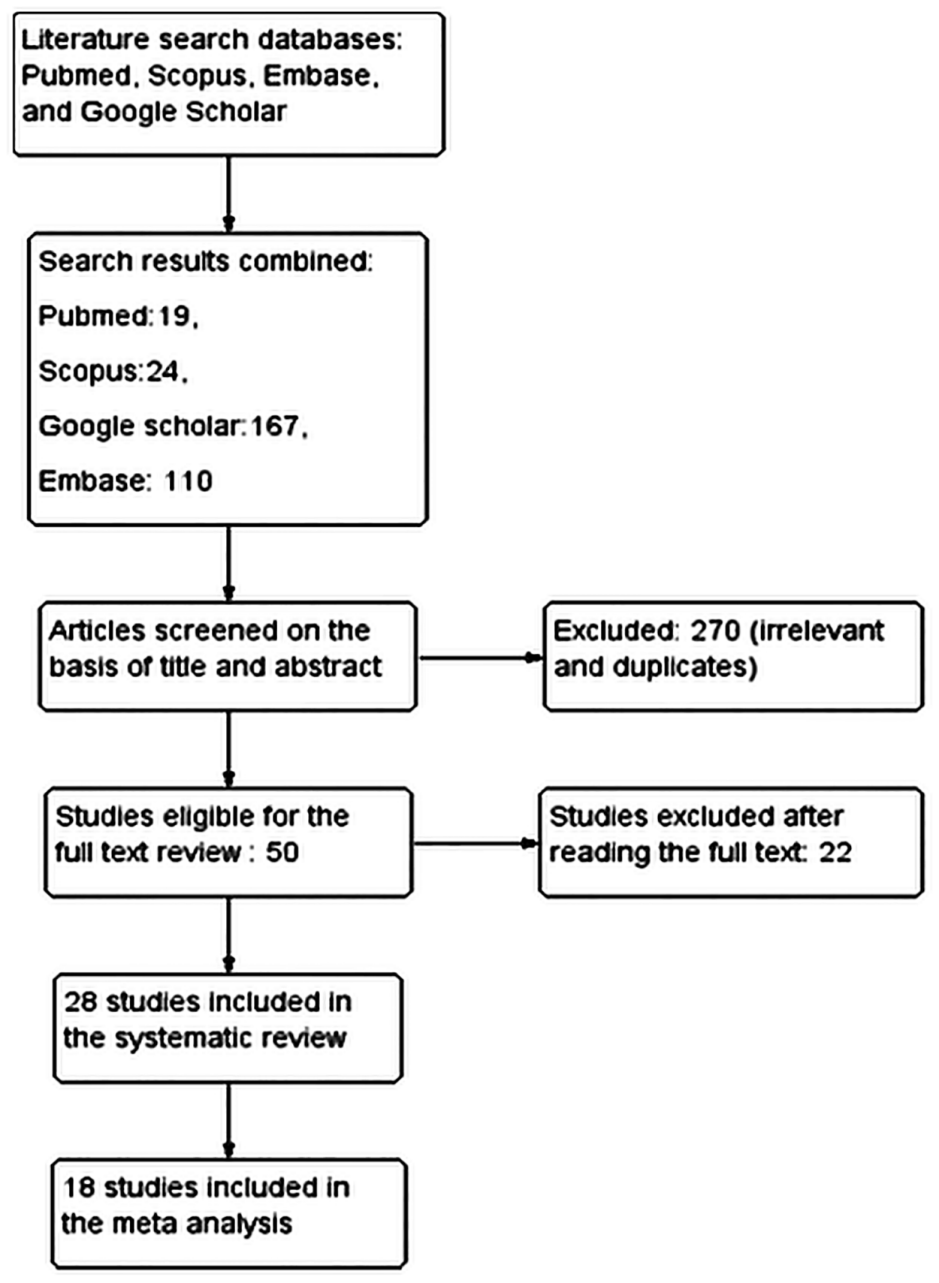
Flow of information through the different phases of the systematic review.

Two authors (ASB and SQJ) analyzed the search results in collaboration to find potentially eligible studies. Small changes in wording were also overlooked for their exact functional meaning. We excluded duplicates and studies in which data was inadequately reported (Fig 1).

### Data extraction

Two researchers independently performed the data extraction and disagreement was resolved by consensus. The extracted data was based on information reported in or calculated from the included studies. Corresponding authors were not contacted for unpublished information. Information including year of execution of the survey, sample size, study location, the method of administration of the questionnaire, and data towards KAP of PV and ADR reporting were retrieved. In particular, we considered six statements that were common to the different studies as outcomes for the meta-analysis:

1. Respondent is aware of the National Pharmacovigilance Programme in India (percentage of inappropriate answers)
2. Respondent considers all drugs to be safe (percentage of inappropriate answers)
3. Respondent is interested in reporting ADRs (percentage of inappropriate answers)
4. Respondent knows where to obtain ADR forms (percentage of inappropriate answers)
5. Respondent never reported an ADR (percentage of yes)
6. Respondent reports non-availability of ADR forms (percentage of yes)

The first two statements assessed the knowledge concerning the national PV programme and safety of drugs, statements three and four assessed the attitude towards ADR reporting, and the last two evaluated the practice of PV activities. Due to differences in the questionnaires administered in different studies, the information suitable to our purpose was extracted for analysis.

### Statistical analysis

Meta-analysis was performed using statistical software StatsDirect 2.8.0 on all the studies that yielded comparable outcomes. Heterogeneity of the studies was evaluated using Cochrane's Q test and the *I*^2^ statistics. Random effects model was used to combine studies showing heterogeneity of Cochrane Q *p*<0.10 and *I*^2^>50% [19]. The methodological quality of studies was assessed using Strengthening the Reporting of Observational Studies in Epidemiology (STROBE) scale [20].

To address the issue of heterogeneity of the studies, a sensitivity analysis was considered using the following subgroups:

- studies of high quality (over 75% of the STROBE checklist)
- studies of low quality (under 75% of the STROBE checklist)

Moreover, publication bias was assessed using Egger and Begg tests and graphs representing funnel plots.

## Results

A total of 320 studies were retrieved from the four scientific databases (Pubmed, Scopus, Embase and Google Scholar) for analysis. After screening titles and abstracts, for duplicates and irrelevant studies, 270 papers were excluded. Fifty studies were considered for the full text review, of which twenty-two were excluded as they did not meet the inclusion criteria (S2 Appendix). Finally, twenty-eight studies [21–48] were selected for the systematic review and eighteen of these were included in the meta-analysis (Fig 1) [31–48].

### Study characteristics

All the 28 citations included in the systematic review were cross-section observational surveys using self-administered questionnaires conducted among Indian healthcare professionals and published between January 2011 and July 2015. Of these, one study was a web-based survey of pharmacists [38]. Eleven studies were conducted in south India [23,24,26–27,29–30, 32–33,40,42], five in the western part of India [22,25,31,45,47], 3 studies in the capital city New Delhi [28,43,48], two in central India [21,37] and one study each was conducted in Bihar [34], Assam [36], Punjab [41], and Jaipur [46]. In addition, three studies did not specify their study location [35,38,44].

The sample size of the studies ranged from 42 [35] to 870 [45] surveyed health professionals. The main characteristics of 28 studies following the STROBE scale are summarized in Table 1. All of the 28 studies met the quality criteria and were selected for systematic review. However, in the meta-analysis, we only included 18 articles (total = 3,187 participants) that are indexed in Pubmed and Scopus that covered all of the main statements.

**Table 1:** Characteristics of Studies included for KAP-PV activities among health professionals in India.

References	Author	Year	Journal	Study design	Study location	Quality assessment*	Sample size	Focusing group	Questionnaire administration	Outcome
	***Studies included for the systematic review***									
[21]	Torwane NA et al	2015	J Nat Accred Board Hosp Healthcare Providers	Cross-section observational study	Bhopal, Madhya Pradesh	-	392	Doctors, dentists, nursing	Close-ended	Knowledge, attitude and practice regarding ADR reporting
[22]	Bansoda AA et al	2015	J Evolution Med Dent Sci	Cross-section observational study	Solapur, Maharastra	>75%	150	Resident doctors	Open and close-ended	Awareness of pharmacovigilance amongst resident doctors
[23]	Sridevi SA et al.	2014	Int J Pharmacol Toxicol	Cross-section observational study	Chennai, Tamil nadu	<75%	324	Medical Postgraduates, graduates, doctors, surgeons, dentists	Close-ended	Knowledge, attitude and practice of ADR reporting and pharmacovigilance
[24]	Kiran LJ et al	2014	Sch J App Med Sci	Cross-section observational study	Karnataka	>75%	120	Clinicians	Close-ended	Knowledge, attitudes
[25]	Karelia BN et al	2014	Int J Basic Clin Pharmacol	Cross-section observational study	Rajkot, Gujarat	<75%	332	Doctors	Open and close-ended	Knowledge, attitudes
[26]	Chanchu S et al	2014	Int J Sci Res	Cross-section observational study	Anantapur, Andhra Pradesh	~ 75%	165	Medical, nursing and pharmacists	Close-ended	Knowledge, attitude and
[27]	Maharani B et al	2013	Nat J Basic Med Sci	Cross-section observational study	Salem, Tamil nadu	>75%	249	Medical Practitioners	Self-administered	Knowledge and attitude of Pharmacovigilance reporting system
[28]	Amrita P et al	2012	Indian J Pharm Prac	Cross-section observational study	New Delhi	<75%	376	Physician, pharmacists, nurses	Close-ended	Knowledge, attitude and skills of pharmacovigilance and spontaneous reporting of ADRs
[29]	Kumar MB et al.	2012	Indian J Pharm Prac	Cross-section observational study	Karnataka	<75%	128	Pharmacists	Close-ended	Knowledge, attitude and behavior of adverse drug reactions reporting
[30]	Kamtane R et al	2012	Asian J Pharm Clin Res	Cross-section observational study	Hyderabad, Telangana	>75%	94	Doctors	Open and close-ended	Knowledge, attitude, and practice of ADR reporting
	***Studies included for systematic review and Meta-analysis***									
[31]	Upadhyaya HB et al	2015	J Adv Pharm Technol Res	Cross-section observational study	Vadodara, Gujarat	>75%	101	Postgraduate Medical students	Open and close-ended	Knowledge, Attitude of Pharmacovigilance and ADRs, and practice towards ADRs
[32]	Gupta SK et al.	2015	Perspect Clin Res	Cross-section observational study	Perambalur, Tamil nadu	<75%	101	Doctors, nurses, and pharmacists	Close-ended	Knowledge, Attitude of Pharmacovigilance and ADRs, and practice towards ADRs
[33]	Ravinadan AP et al	2015	Asian J Pharm Clin Res	Cross-section observational study	Davangere, Karnataka	<75%	102	Pharmacists	Close-ended	Knowledge, attitude, and practice of pharmacists towards adverse drug reaction (ADR) reporting
[34]	Panja B et al	2015	Indian J Pharmacol	Cross-section observational study	Kishanganj, Bihar	-	66	Postgraduate medical students	Open and close-ended	Awareness of pharmacovigilance and ADR reporting
[35]	Aithal S et al.	2014	Int J Pharm Bio Sci	Cross-section observational study	Unspecified	>75%	42	Doctors	Open and close-ended	Knowledge, attitude, and practice of ADR reporting
[36]	Gupta P et al.	2014	Mal Res Treat	Cross-section observational study	Assam	<75%	154	Health care professional	Close-ended	Knowledge and attitude of Pharmacovigilance Practice
[37]	Khan SA et al	2013	J Nat Sci Biol Med	Cross-section observational study	Indore, Madhya Pradesh	<75%	68	Doctors	Open and close-ended	Awareness and knowledge of ADR reporting, attitude and practice of ADR reporting
[38]	Ahmad A et al	2013	Perspect Clin Res	Cross-sectional web-based survey	Unspecified	<75%	400	Pharmacists	Open and close-ended	Knowledge, attitude, and practice of ADR reporting
[39]	Thomas TM et al	2013	Int J Pharmacol Clin Sci	Cross-section observational study	Manglore, Karanataka	-	60	Doctors	Close ended	Knowledge, attitude, and practice of ADR reporting
[40]	Choudary AK et al.	2013	Internet J Pharmacol	Cross-section observational study	Trichy, Tamil nadu	>75%	121	Doctors, nurses, pharmacists	Open and close-ended	Knowledge, attitude and practice of ADR reporting
[41]	Hardeep L et al	2013	J Clin Diagnos Res	Cross-section observational study	Jalandhar, Punjab	<75%	61	Doctors	Open and close-ended	Awareness of pharmacovigilance
[42]	Prakasam A et al	2012	Pharm Prac	Cross-section observational study	Hyderabad, Telangana	<75%	347	Community Pharmacy	Close-ended	Knowledge about Pharmacovigilance, Perception and practice towards ADR reporting
[43]	Rehan HS et al	2012	Indian J Pharmacol	Cross-section observational study	New Delhi	>75%	200	Resident doctors and Nurses	Open and close-ended	Knowledge of adverse drug reactions, attitude and practice of ADR monitoring and reporting
[44]	Pimpalkhute SA et al	2012	Indian J Med Sci	Cross-section observational study	Unspecified	>75%	84	Resident doctors	Open and close-ended	Knowledge and attitude towards ADR reporting
[45]	Kharkar M et al.	2012	Perspect Clin Res	Cross-section observational study	Mumbai, Maharastra	>75%	870	Medical Practitioners	Open and close-ended	Knowledge, attitude and practice of ADR reporting
[46]	Upadhaya P et al.	2012	Ther Clin Risk	Cross-section observational study	Jaipur, Rajasthan	-	50	Postgraduate doctors	Open and close-ended	Knowledge of ADR reporting
[47]	Desai CK et al.	2011	Perspect Clin Res	Cross-section observational study	Ahmadabad, Gujarat	<75%	260	Consultants, postgraduates, resident doctors	Open and close-ended	Knowledge regarding ADR reporting system, Attitude and Practice of ADR reporting.
[48]	Chopra D et al	2011	Int J Risk Saf Med	Cross-section observational study	New Delhi	-	100	Doctors	Open and close-ended	Knowledge, attitude and practice of ADR reporting and Pharmacovigilance

Systematic review includes 28 studies, N = 5517; Meta-analysis -18 studies, N = 3187.

* Quality of the study assessed using the "STROBE Statement checklist".

J Nat Accred Board Hosp Healthcare Providers- The Journal of National Accreditation Board for Hospitals & Healthcare Providers; J Evolution Med Dent Sci- Journal of Evolution of Medical and Dental Sciences; Int J Pharmacol Toxicol-International Journal of Pharmacology and Toxicology; Sch J App Med Sci- Scholars Journal of Applied Medical Sciences; Int J Basic Clin Pharmacol- International Journal of Basic and Clinical Pharmacology; Int J Sci Res- International Journal of Science and Research; Nat J Basic Med Sci- National Journal of Basic Medical Sciences; Indian J Pharm Prac-Indian Journal of Pharmacy Practice; Asian J Pharm Clin Res- Asian Journal of Pharmaceutical and Clinical Research; J Adv Pharm Technol Res- Journal of Advanced Pharmaceutical Technology and Research; Perspect Clin Res- Perspectives in Clinical Research; Indian J Pharmacol- Indian Journal of Pharmacology; Int J Pharm Bio Sci- International Journal of Pharma and Bio Sciences; Mal Res Treat- Malaria Research and Treatment; J Nat Sci Biol Med- Journal of Natural Science, Biology and Medicine; Int J Pharmacol Clin Sci- International Journal of Pharmacology and Clinical Sciences; Internet J Pharmacol- Internet Journal of Pharmacology; J Clin Diagnos Res- Journal of Clinical and Diagnostic Research; Pharm Prac- Pharmacy Practice (Granada); Indian J Med Sci- Indian Journal of Medical Sciences.

### Study results and meta-analysis

The number of inappropriate responses was considered for each statement regarding KAP of PV and ADR reporting.

#### Knowledge about PV and Drug safety in India

**Fig 2:**
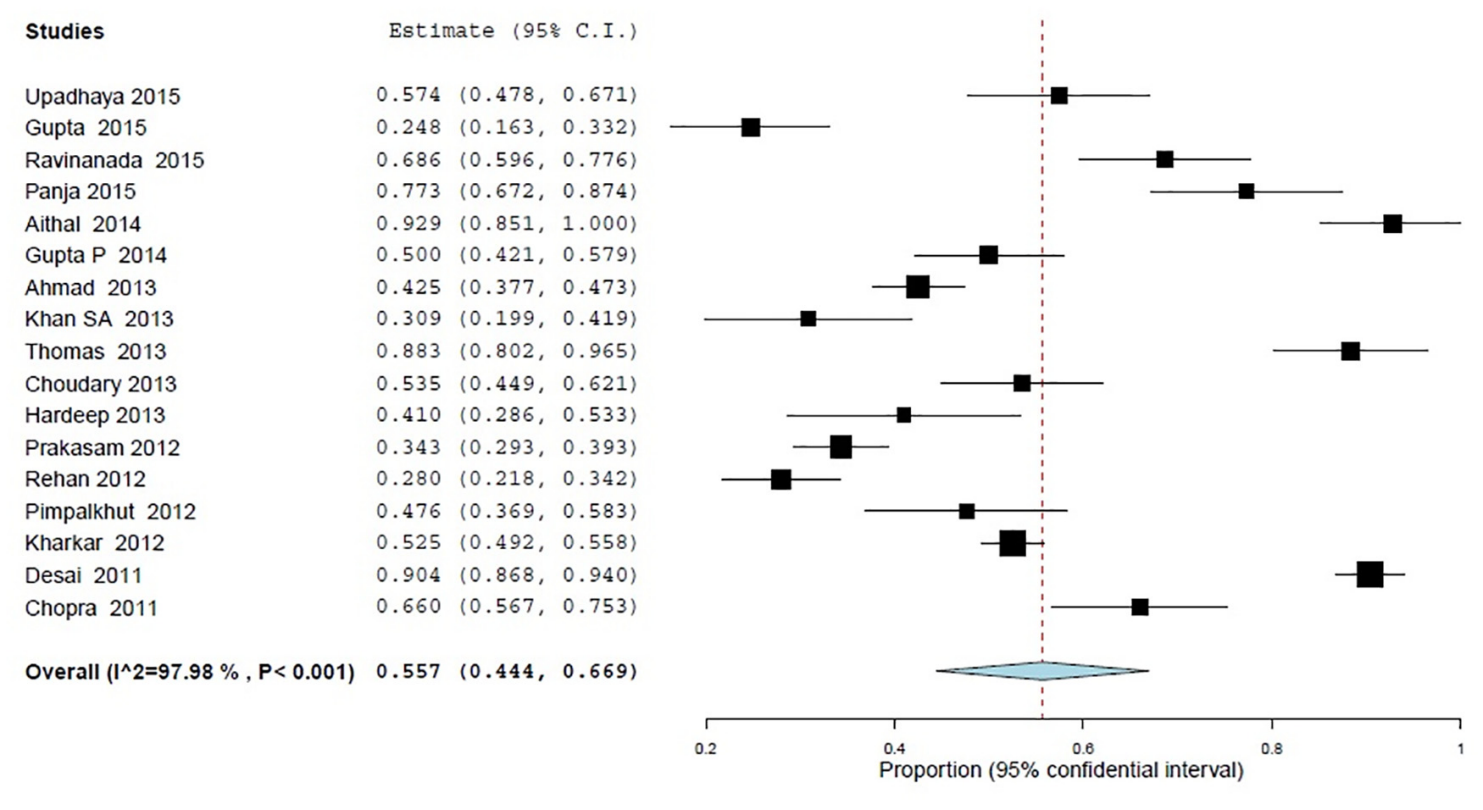
Awareness of existing Pharmacovigilance programme in India (PVPI), (% of inappropriate answer) 17 studies, N = 3145.

**Fig 3:**
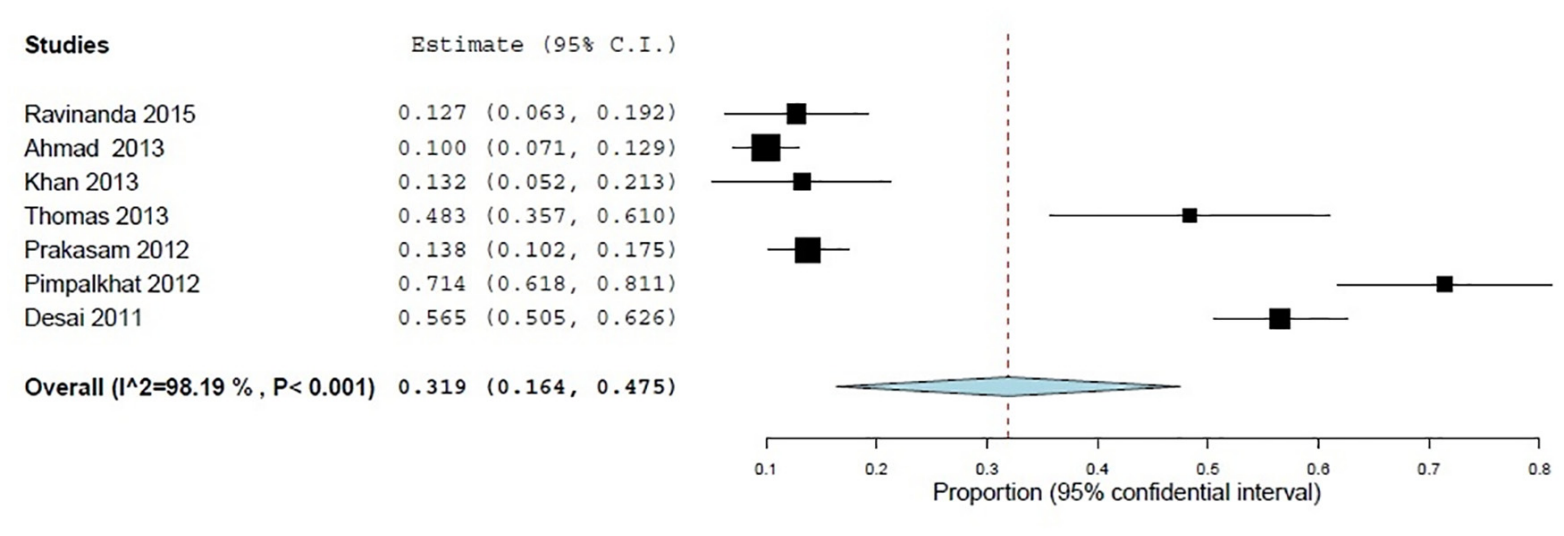
Respondents considered all drugs available in market are safe, (% of inappropriate answers) 7 studies, N = 1321.

Two statements were used in the assessment of knowledge regarding the awareness of existing PvPI and safety of drugs. Overall, 55.6% (95% CI 44.4–66.9; *p*<0.001) of the health professionals gave an incorrect answer to the statement "Are you aware of the existence of national pharmacovigilance programme in India" (Fig 2). In addition, 31.9% (95% CI 16.3–47.4; *p*<0.001) of the sample erroneously thought that "all drugs available in the market are safe" (Fig 3).

#### Attitude of health professionals

**Fig 4:**
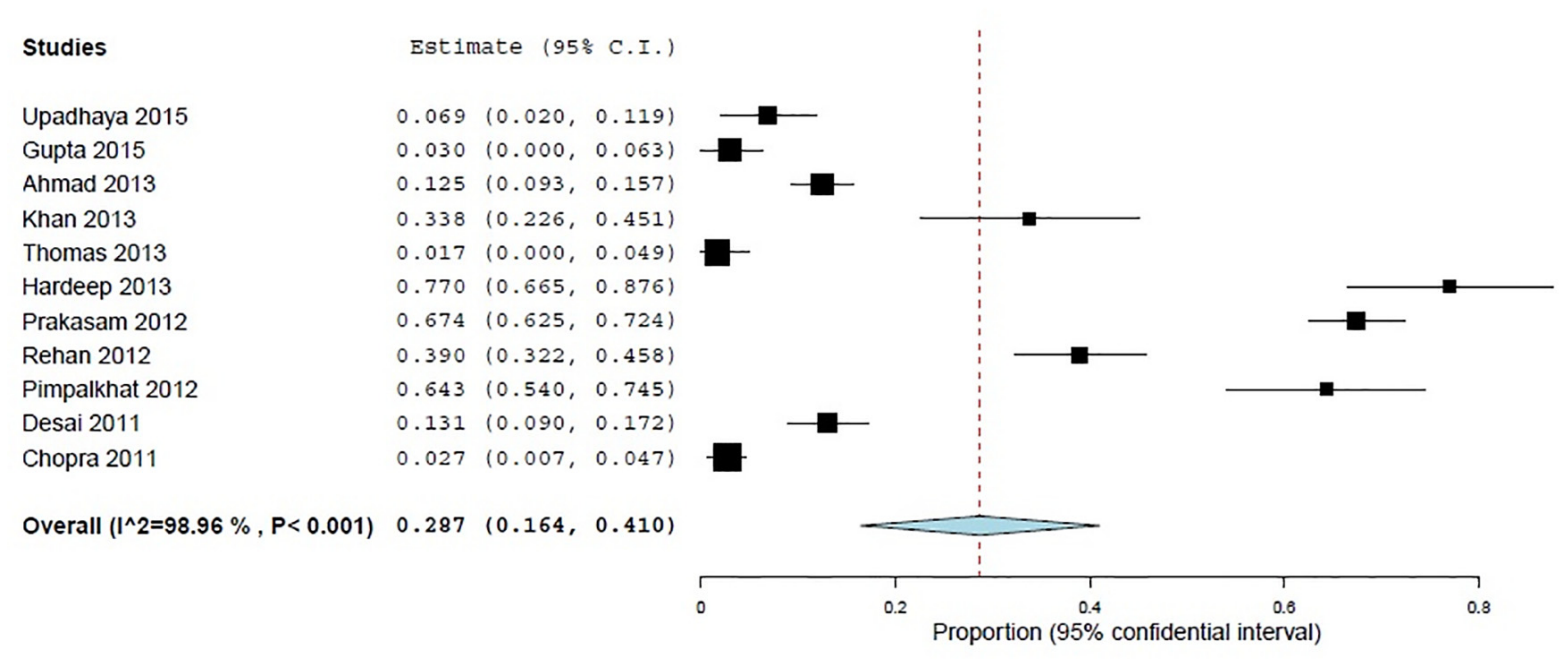
Respondents interest to report ADRs, (% of inappropriate answers) 11 studies, N = 1942.

**Fig 5:**
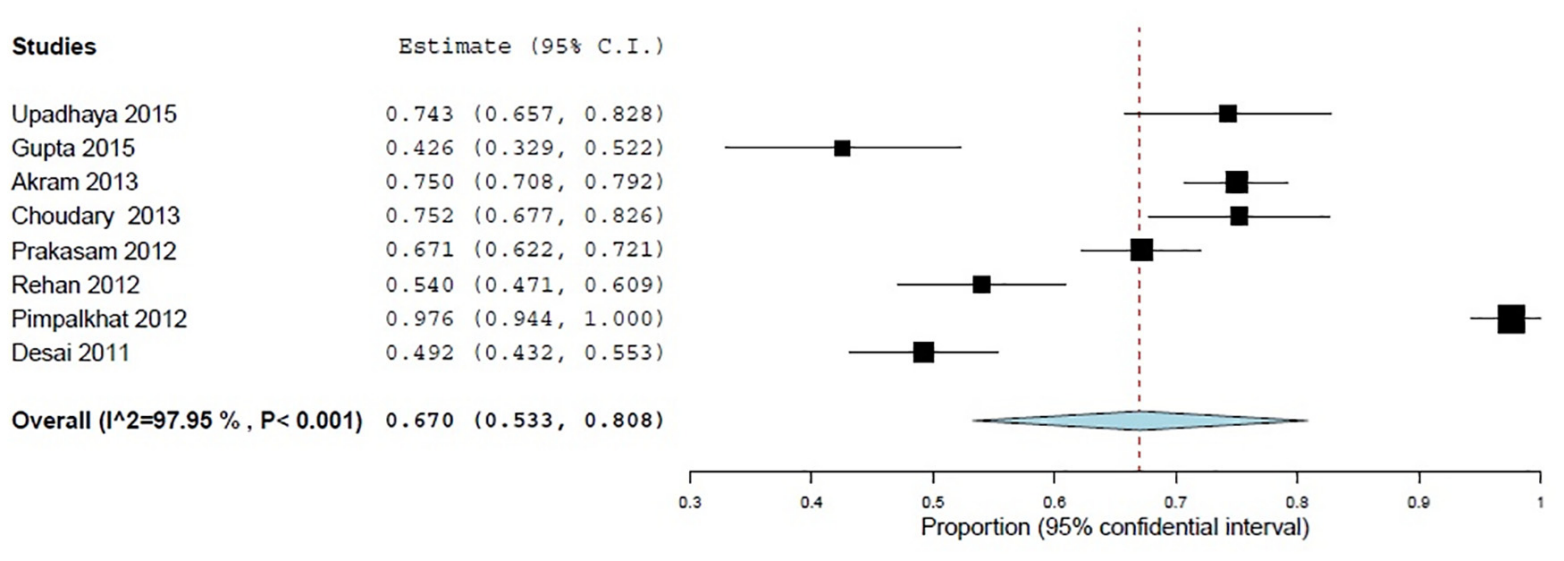
Respondents know where to obtain ADR forms, (% of inappropriate answers) 8 studies, N = 1622.

Attitude towards reporting ADRs were assessed using two statements. 28.7% (95% CI 16.4–40.9; *p*<0.001) participants declared that they were not interested in reporting ADRs to PV centers and 67% (95% CI 53.2–80.8; *p*<0.001) commented that they did not know where to obtain ADR reporting forms (Figs 4 and 5).

#### ADR reporting practices

**Fig 6:**
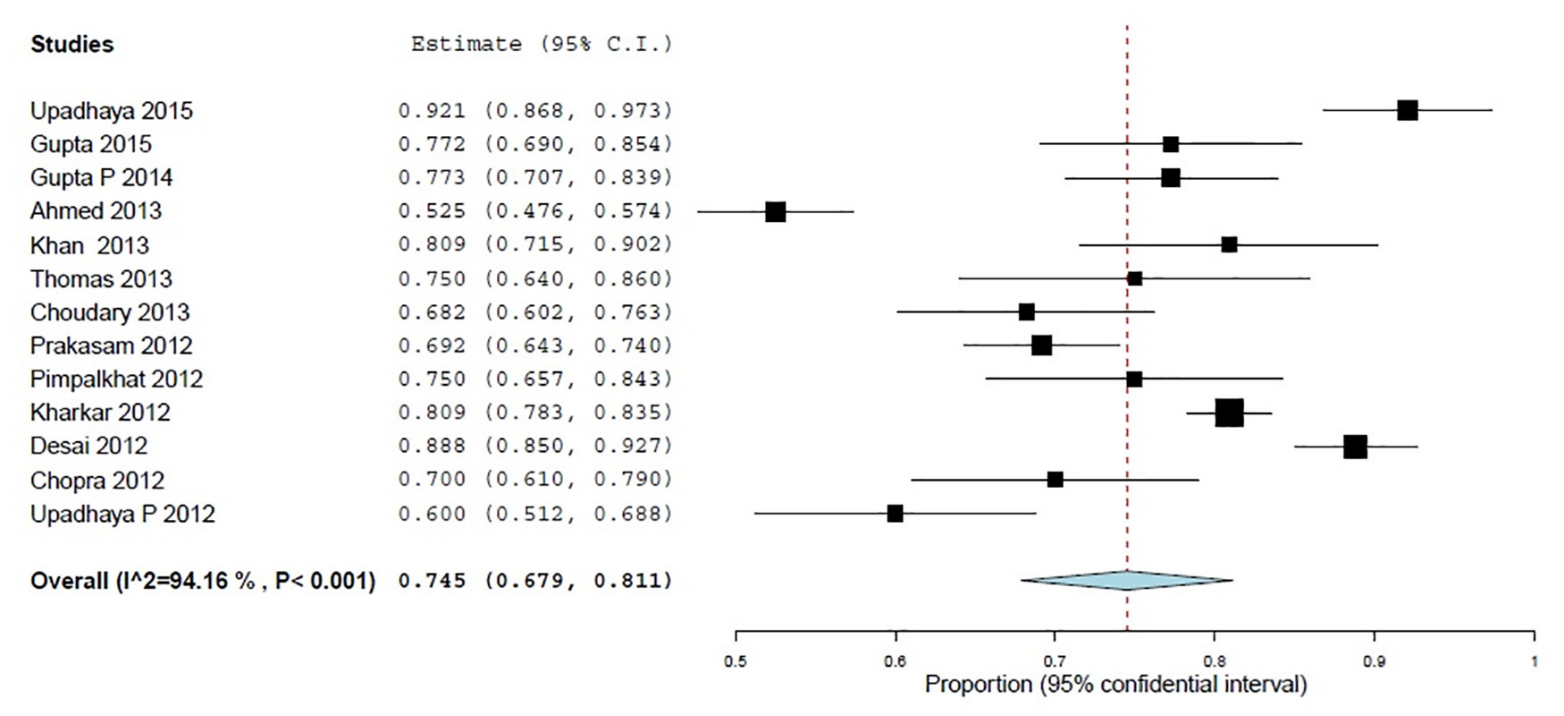
Never reported an ADR (% of Yes) 13 studies, N = 2794.

**Fig 7:**
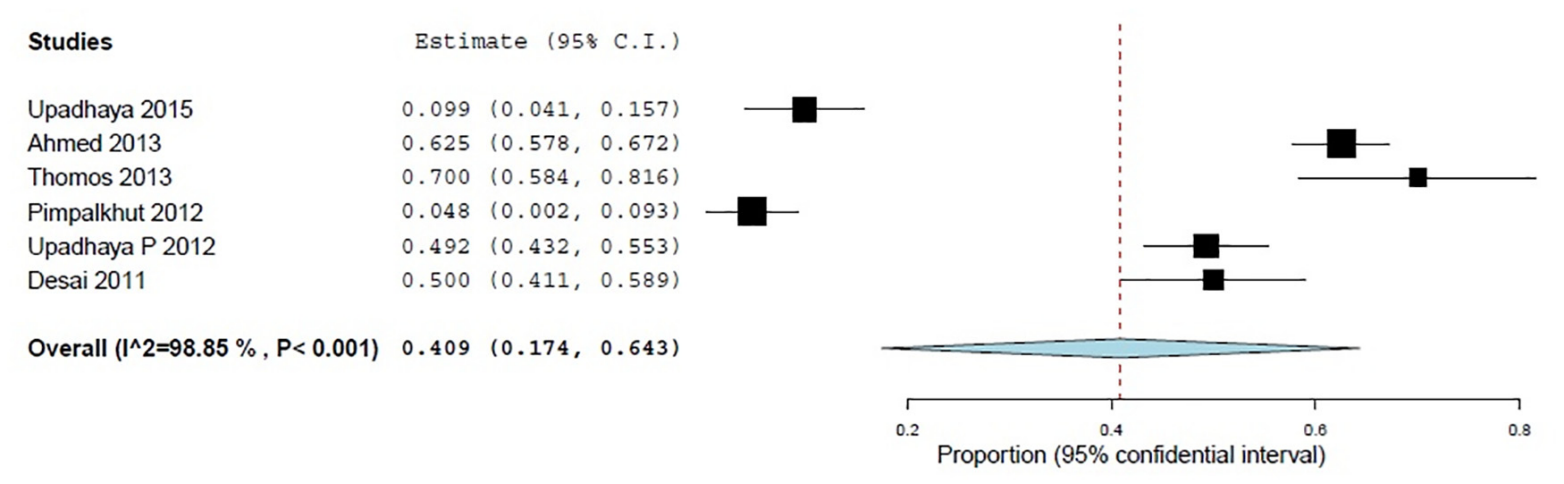
Non-availability of ADR forms (% of Yes) 6 studies, N = 1025.

To the statement "Had you ever reported an ADR to a PV center" nearly three-quarters (74.5%, 95% CI 67.9–81.9; *p*<0.001) of the sample declared that they never reported any ADR to PV centers and 40.8% (95% CI 17.4–64.3; *p*<0.001) of them ascribed it to "non-availability of ADR forms at their sites" (Figs 6 and 7).

### Publication bias

Publication bias was not highlighted in any of the 18 studies analyzed. Based on Egger and Begg tests graph confirmed by the funnel plot.

### Sensitivity analysis

Meta-analysis was stratified based on the quality of the studies (high and low quality) to reveal the KAP concerning PV activities in India. For the statement "Are you aware of the existence of PvPI" the percentage of high-quality studies incorrectly answered 55.3% (95%CI 39.2–71.2) compared to 53.5% (95%CI 37.8–69.1) low-quality studies. Furthermore, sensitivity analysis of the low-quality studies showed a negative attitude of 30.8% (95%CI 10.5–51.1) towards reporting ADRs as compared to the non-stratified groups. However, failure to report ADRs among health professionals was 76.7% (95%CI 64.0–89.3), higher than in the main analysis in high-quality studies.

## Discussion

This systematic review was aimed at assessing the KAP towards PV and ADR reporting in studies conducted in India during January 2011 to July 2015. To the best of our knowledge, this is the first systematic review and meta-analysis on this topic. In 2009, two reviews focused on under-reporting of ADRs [49] and PV steps in causality assessments [50].

### Strengths and limitations

Our study is the first to execute the KAP of PV activities in India by including 18 studies in meta-analysis. This has helped us gather and strengthen the combination of each study results, providing stronger evidence about ongoing PV activities among Indian health professionals. Our search strategy was comprehensive, including studies published in English, and research using standard questionnaires conducted in India.

### Summary of study findings

We found that more than 50% of the sample were not aware of PvPI and around 32% thought that all drugs available in market were safe (allopathic, herbal/traditional, blood products, biological and medical devices). Although 71.3% were interested in reporting suspected ADRs, 67% did not know where to obtain ADR reporting forms. Indeed, three-quarters of the sample never reported an ADR to any PV centers. Additionally, 40.8% declared that ADR reporting forms were not readily available at their sites to enable ADRs reporting. Overall, we found that more than 40% of the sample have demonstrated inadequate knowledge, attitude and more than half never actually reported an ADR to the PV centers.

The findings of our review identified the lack of knowledge regarding PV and drug safety in India. For instance, nearly 55% of the population answered incorrectly regarding the existence of PvPI in India. This is in agreement with the recent research conducted on 90 ADR monitoring centers working under PvPI which highlighted that 68% of the doctors, 80% of nurses and 81% of the pharmacists are unaware of PvPI in India [12]. Lack of awareness regarding the existence of PvPI may be an important deterrent to ADR reporting. In *Bisht et al* [51] intervention study, more than 50% of the CME (continuous medical education) attended doctors were unaware of existence of PvPI. Interestingly, doctors attending CME showed a higher resistance toward ADR reporting than non-CME attendees (57% versus 33%). To improve awareness among health professionals, various approaches were attempted during the past three years that include awareness lectures, public campaigns, conferences, workshops, post-training reminders such as periodic E-mails and SMS alerts [52].

The health professionals’ lack of knowledge regarding safety of marketed drugs could lead to serious drug reactions in patients. Remedies such as allopathic, ayurveda, homeopathy, unani, and other therapies are widely practiced in India. In 2012, the South Asian Association for Regional Cooperation (SAARC) reported that there are more than 4246 herbal drugs registered and available as over-the-counter without restrictions in India [53]. Furthermore, concerted use of allopathic and non-allopathic medications poses the risk of serious drug-drug interactions that may produce potential adverse outcomes. Hence, it is essential for all health professionals to study the safety profiles of medications before prescribing them and be vigilant in reporting any suspected ADRs to the PV centers. Reporting suspected ADRs for medications promotes a deeper understanding of their safety profile in a real clinical settings.

Further, it was clearly observed that a part of the health professionals (28.7%) were not interested in reporting suspected ADRs. This attitude showcases the passive perception of some of the health professionals ignoring the importance of reporting ADRs. Evidence from various national and international studies suggested that lethargy, diffidence, insecurity and overwork were some of the factors for under-reporting of ADRs by health professionals [12,49]. In particular, *Desai and co-workers*, described that 70% of their study participants did not know where to report the suspected ADRs [47]. Other studies also identified other reasons such as 'unreported could not make any difference' [51], 'lack of financial incentives' [30,38], 'ignorance (that only serious ADRs are to be reported)' [54] and 'lack of time' [55,56]. It is therefore necessary to motivate the health professionals by repetitive educational interventions and simplification of the ADR reporting process which might encourage health professionals to report ADRs in India. Importantly, making ADR reporting online is also recommended based on our findings.

Practice of PV is crucial for generating a national safety database of drugs. Our meta-analysis identified that a majority of the health professionals never reported any ADR encountered during their practice. The average Individual Case Safety reports (ISCRs) received per month by Vigiflow from 12 PV centers during the period of 2011 to 2013 was 48.3 and the rate of spontaneous reporting was only 33.8%. There are manifold hindering factors that need further investigation to understand the barriers influencing the practice of PV. Indeed, it is a difficult task to foster PV practice culture without proper knowledge and attitude. Possible solutions for improving PV practice in India might be implementing strong regulations to report ADRs after providing sufficient training to the health professionals and simplifying the process of ADR reporting using electronic system by giving some amount of financial incentives to health professionals.

### Limitations

This study has some limitations that should be considered. Our meta-analysis showed some heterogeneity with a consistent lack of homogeneity of the responses. This heterogeneity could be due to sociodemographic, inter-professional, and cultural variations among the health professionals across India. Moreover, there were differences in sample selection and the way questionnaires were administered that may lead to selection bias. In addition, questionnaires administered in all studies were open and closed-ended and the population studied may have overestimated or underestimated when responding which may have led to recall bias. Quality assessment and stratification of quality and geographic criteria have allowed the evaluation of the presence of potential bias and confounding. For instance, poor quality could influence the KAP regarding the real function of PV in India.

## Conclusion

Our results identified a huge-gap pertaining to KAP towards PV activities in India. The fact that more than 75% of the health professionals never reported an ADR, raises questions on PV activities in India. Educational campaigns and training to improve the knowledge; financial incentives and simplifying the reporting process might change the attitudes. Further, making ADR reporting mandatory can make health professionals aware of the importance of PV in India. In order to better understand the PV progress, PvPI should periodically assess the KAP of health professionals PV activities in India.

## Supporting Information

S1 Appendix PRISMA 2009 Checklist. (DOCX)

S2 Appendix Supporting Information Data. (DOCX)
