# Supplementary material for: Health Professionals' Knowledge, Attitudes and Practices about Pharmacovigilance in India: A Systematic Review and Meta-Analysis
Source: PLoS One. 2016 Mar 24;11(3):e0152221. doi: 10.1371/journal.pone.0152221 (PMC4807086; doi:10.1371/journal.pone.0152221)
Supplement: S2 Appendix — (DOCX) [file pone.0152221.s002.docx]

**List of Excluded studies**

1. Bisht M, Singh S, Dhasmana DC. Effect of educational intervention on adverse drug reporting by physicians: A cross-sectional study. ISRN Pharmacol 2014; Article ID: 259476 *(not eligible:not a cross-sectional study)*

2. R. Radhakrishnan R, Sudha V, Danturulu MV. An educational intervention to assess knowledge attitude practice of pharmacovigilance among Health care professionals in an Indian tertiary care teaching hospital,” Int J PharmTech Res 2011;3(2):678–692. *(not eligible, not a cross-sectional study)*

3. Tandon VR, Mahajan V, Khajuria V, Gillani Z. Underreporting of adverse drug reactions: A challenge for pharmacovigilance in India. Indian J Pharmacol 2015;47(1):65-71. (*not enough information)*

4. Rajakannan T, Mallayasamy S, Guddattu V, Kamath A, Vilakkthala R, Rao PG, et al. Cost of adverse drug reactions in a south Indian tertiary care hospital. J Clin Pharmacol 2012;52(4):559-565. *(not eligible: out of study objective scope)*

5. Khan LM. Compartive epidemiology of hospital-acquired adverse drug reactions in adult and children and their impact on cost and hospital stay-a systematic review. Eur J Clin Pharmacol 2013;69(12):1985-1996 *(not eligible; out of study objective scope)*

6. Choudhary AK, Nivedhitha S, Ramakrishna L, Tirumalaikolundusubrimnian P, Manicvasagam S. Perception of adverse drug reaction among doctors, nurses and pharmacists of a tertiary care rural teaching hospital. Internet J Pharmacol 2013;12(1):1566. *(not eligible; not following structured questionnaire)*

7. Sanghavi DR, Dhande PP, Pandit VA. Perception of pharmacovigilance among doctors in a tertiary care hospitals: influence of an interventional lecture. Int J Risk Saf Med 2012;25(4):197-204. *(not eligible: not a cross-sectional study)*

8. Rishi RK, Patel RK, Bhandari A. Under reporting of ADRs by medical practitioners in India - results of pilot study. Adv Pharmacoepidem Drug Saf 2012;1(3):112 *(not eligible; not following structured questionnaire)*

9. Chetty S, Amrita P, Shalini A, Bairy KL. Knowledge, attitude and practice of healthcare professionals towards adverse drug reaction reporting in a South Indian teaching hospital. World J Pharmaceutical research 2014;3(3):4263-4271 *(results were not adequately presented)*

10. Mishra H, Kumar V. Pharmacovigilance: current scenario in a tertiary care teaching medical college in north India. J Pharmacovigil 2013;1(2):108. *(not enough information)*

11. Reddy VL, Pasha SJ, Rathinavelu M, Reddy YP. Assessment of knowledge, attitude and perception of pharmacovigilance and adverse drug reaction (ADR) reporting among the pharmacy students in south India IOSR J Pharm Bio Sci 2014;9(2):34-43 *(not eligible; not following structured questionnaire)*

12. Jagminder KB, Kumar R. A survey on the knowledge, attitude and the practice of pharmacovigilance among the health care professionals in a teaching hospital in northern India. J Clin Diagnos Res 2013;7(1):97-99 (*data overlap with Hardeep et al. paper*)

13. Sengupta G, Bhowmick S, Hazra A, Dutta A, Rahaman M. Adverse drug reaction monitoring in psychiatry out-patient department of an Indian teaching hospital. Indian J Pharmacol 2011;43(1):36. *(not eligible: out of study objective scope)*

14. Elizabeth ST, Kia RA, Yagnik RM, Nagaraju K. Knowledge, attitude and skills of nurses of Delhi towards adverse drug reaction reporting. Indian J Pharm Prac 2012;5(1):45-51 *(not eligible: results were not relevant with the title)*

15. Vora MB, Paliwal NP, Doshi VG, Barvaliya MJ, Tripathi CB. Knowledge of adverse drug reaction and pharmacovigilance activity among the undergraduate medical students of Gujarat. Int J Pharm Sci Res 2012;3(5):1511 *(data errors in the results)*

16. Praveen S, Prakash R, Manjunath GN, Gautham MS, Kumar N. Adverse drug reaction reporting among medical and dental practitioners: a KAP study. Indian J Med Special 2013;4(1):10-15 *(data errors in the results)*

17. Kalaiselvan V, Prasad T, Bisht A, Singh S, Singh GN. Adverse drug reaction reporting culture in pharmacovigilance programme of India. Indian J Med Res 2014;140:563-4. *(not a original article)*

18. Hema NG, Bhuvana KB, Sangeetha KB. Pharmacovigilance: The extent of awareness among the final year students, interns and postgraduates in government teaching hospital. J Clin Diagnos Res 2012;6(7):1248-1253 *(results were differently presented)*

19. Ahmed A, Patel I, Sanyal S, Balkrishnan R, Mohanta GP. A study on drug monitoring program in India. Indian J Pharm Sci 2014;76(5):379-86 *(not eligible: out of study scope)*

20. Adhikary J, Bhandare B, Adarsh E, Satyanarayana V. A study to assess knowledge, attitude, and practice of adverse drug reaction reporting among physicians in a tertiary care hospital. J Evolution Med Dent Sci 2013;2(9):1027-1034 *(data errors in the results)*

21. Remesh A. Identifying the reasons for under reporting of ADR: A cross sectional survey. Res J Pharm Bio Chem Sci 2012;3(4):1379-1386 *(not eligible: out of study scope)*

22. Nirojini PS, Murrapu SP, Sanaka M, Nadendla RR. A survey on assessing the knowledge, attitude and behavior of pharmacy teachers to adverse drug reaction related aspects. Indo American J Pharm Res 2015;5:8-14 *(not eligible: not used structured questionnaire)*
